# The Food Identity of Countries Differs Between Younger and Older Generations: A Cross-Sectional Study in American, European and Asian Countries

**DOI:** 10.3389/fnut.2021.653039

**Published:** 2021-08-16

**Authors:** Lucía Frez-Muñoz, Jarl K. Kampen, Vincenzo Fogliano, Bea L. P. A. Steenbekkers

**Affiliations:** ^1^Food Quality and Design Group, Wageningen University & Research, Wageningen, Netherlands; ^2^Mathematical and Statistical Methods–Biometris, Wageningen University & Research, Wageningen, Netherlands

**Keywords:** traditional food and beverage, consumer perception, age cohort, food identity, culture

## Abstract

Current generations have been strongly influenced by mass communication and massive immigration flows, which may change local lifestyles and perceptions of inhabitants towards traditional foods and beverages (TFBs). TFBs constitute a core element of the cultural identity of a country, although some of them are losing their appeal. In this study, the authors explored the TFBs perceptions of inhabitants in nine countries to determine if their food identity is changing by analysing the TFBs most frequently mentioned by different age cohorts within a country. Six countries were found to have a distinctive core of TFBs shared across age cohorts (homogenous), whereas the remaining showed a heterogeneous pattern. Correspondence and cluster analyses usually grouped younger generations together implying higher similarities among these cohorts. Furthermore, the binary logistic regression analyses performed identified significant differences in the probability of mentioning a specific TFBs across age cohorts per country. Data collected show younger cohorts focusing on TFBs categorised as snacks and foods on-the-go, whereas older cohorts more often refer to time-consuming savoury preparations. The results suggest that lifestyles and current societal trends in food consumption, for example, convenience and healthiness, are impacting the food culture and identity of countries, and therefore play an important role in the variation of TFBs perception and consumption between age cohorts within countries. The results obtained in this study could not only be used by food policymakers and nutritionists to distinguish the current trends that are reshaping the food identity and eating behaviours of the population but also to improve or develop new dietary strategies by age cohorts in the countries studied.

## Introduction

The foods consumed by a person not only provide nutrition but also define his/her identity. Two dimensions have been described to represent this relationship, namely from biological to cultural (nutrition to symbolic functions) and from individual to collective (phycological to collective functions) (1). In this context, some foods may also provide symbolic functions in a culture, as is the case of traditional foods and beverages (TFBs). Thus, the spectrum of TFBs perceived as such and consumed by inhabitants define the food culture of a specific country. TFBs have been inherited through generations, making them the representative of the cuisine of the country, and therefore a faithful estimation of the eating habits followed by its inhabitants (2). However, countries and their cuisines have changed over time influenced by several historical phenomena, as described below.

Since the early years, relevant historical phenomena such as colonialism have impacted countries by reshaping geographical boundaries, restructuring cultures and lifestyles, and increasing the accessibility of ingredients (3, 4). Trade allowed the cross-continental introduction of foreign ingredients, for example, tomatoes and potatoes from America and spices from the Middle East, having a strong effect on the composition of TFBs all over the world (5). Similarly, globalisation is continuously fostering immigration and the diversification of culinary repertoires by facilitating the flow of foods and information available worldwide (6–8).

Several milestones across the lifespan of the consumers, e.g., entering the workforce, living on your own, and having children, condition food choice and behaviour (9, 10). Nonetheless, the interplay between the lifestyles of the consumers, family culinary traditions, and the food culture of a country strongly influences the assortment of (traditional) foods being consumed, and perceived as TFBs by different age groups.

In the last decades, researchers have placed more attention on the study of traditional foods to understand the eating habits of consumers to improve the dietary guidelines and policies of countries (2). Previous studies related to traditional foods developed a definition of traditional food products from the perspective of a consumer (11), determined the motives for choosing and consuming TFBs in Europe (12), unravelled the perceptions and preferences of consumers for specific TFBs (13) and studied the impact of innovations in TFBs (14, 15). However, which food products are perceived as traditional by inhabitants of different countries and to what extent the age of a consumer exerts an effect on this perception was not investigated. Hence, here we aimed to determine if the food identity differs among age cohorts in different countries by studying the similarities and differences in the foods and beverages perceived as traditional between generations in nine countries (The Netherlands, Italy, Hungary, Brazil, Chile, Mexico, Indonesia, China, and Japan). In addition, we investigated the types of foods (dish, snack, or beverages) and the consumption moment (regular basis, special occasions/celebrations, and/or seasonally) to compare several aspects that may be influencing the perceptions of consumers from different cultural backgrounds and dietary habits.

## Materials and Methods

### Research Instrument and Sample

A questionnaire was designed by the authors and the study was approved by the Social Sciences Ethics Committee at Wageningen University & Research to determine the TFBs in Mexico, Brazil, Chile, The Netherlands, Hungary, Italy, China, Japan, and Indonesia. The countries were chosen based on two aspects, which are (i) their geographical distribution within a continent, to cover different countries within a region/continent; and (ii) the background of their food culture. The TFBs in a country have been influenced by several forces, e.g., colonisation, trade, and the rearrangements of nations being some of them (3–8). Thereby, countries that were connected through colonisation were also selected. For instance, Brazil was included since it has a different background than the other countries of that region due to the African and Japanese immigrants (16, 17). Indonesia was colonised by The Netherlands; therefore, some level of overlap in the foods and beverages mentioned was expected (18). Adult inhabitants (≥18 years of age) who were born and raised in each country were part of this research to ensure they were highly familiar with their traditional cuisine.

The validity of the questionnaire was tested through content validity to determine whether the research instrument was measuring what was intended (19). The food quality and design group at Wageningen University is composed of researchers who work in several topics linked to consumer studies, food quality management, system dynamics, food safety, food quality modelling, design of healthy foods, etc., who come from several nationalities, including the ones selected for this study. Therefore, staff members, PhD candidates, MSc, and BSc students were kindly asked to take a pilot survey as if they were in their country of origin to test the survey and if the constructs were well formulated. At the end of the survey, a special box was left open for them to provide comments and suggestions, and also barriers that they may have encountered. After the first pilot, the questions were adjusted, when necessary, and another pilot was carried out to crosscheck the constructs and final details, such as ease of answering the survey when using different devices (laptop or mobile) and length of the survey. Regarding the languages, the questionnaire was developed in English and translated into the mother tongues by two natives. If differences were found, which rarely happened, a third person was asked to back-translate the sentences that were different in order to keep the one that was in line with the English version.

The surveys were transferred to the Qualtrics Software (20) and spread through the snowball sampling method and *via* social media in 2018. They were divided into demographic questions and core-questions addressing the main aspects to be investigated, namely: (i) listing 10 dishes/snacks/beverages perceived as traditional by following this definition: “*Traditional foods and beverages have a strong connectionto the cultural identity of a country and have passed through generations; hence they are representative of its local cuisine. Most importantly, they are perceived as such by inhabitants, known and still consumed nationwide*”; (ii) classifying the TFBs into six categories namely dishes (savoury/sweet), snacks (savoury/sweet), and/or beverages (alcoholic/non-alcoholic). Snacks were defined as “*A small amount of food that is eaten between meals, or a very small meal”*; and (iii) stating the consumption moment: regular basis, special occasions/celebrations and/or seasonally. As incentive, respondents from each country could participate in one out of four gift cards worth €10. Within countries, sampling continued until the point where saturation was reached regarding the TFBs mentioned by the respondents, that is, when new incoming data no longer appended the list of already mentioned TFBs.

### Data Preprocessing

The data gathered was preprocessed in order to ensure consistency in the responses collected across the countries. Two stages were followed during the preprocessing of the data. First, only the answers of respondents who completed the survey were included in the analyses. The average response rate across countries was 66%, 10 min being the average response time. Second, five criteria were developed to systematically distinguish the TFBs mentioned within a country (Table 1) and used by two to three natives from each country in the analyses.

**Table 1:** List of criteria used for the operationalisation of the traditional foods and beverages concept.

**Inclusion criteria**	**Explanation**
1. Is representative of a country's cuisine	Foods and beverages that are in line with the definition: ‘*Traditional foods and beverages have a strong connection to the cultural identity of a country and have passed through generations; hence they are representative of its local cuisine. Most importantly, they are perceived as such by inhabitants, known and still consumed nationwide*'.
2. Is nationally available^*^	We aimed at having a representative sample of each country's cuisine by considering traditional foods that were available nationwide. ^*^However, in those countries that are divided into tribes and/or islands, e.g., Indonesia, this condition was flexible only when the food was the same (or similar), but has different names. In this case, they were grouped together.
3. Is a processed dish, snack or beverage	Some form of processing was expected, e.g., roasted, oven baked, boiled, etc.
4. Is not an ingredient or basic product^*^	Ingredients or basic products commonly present around the world such as apple, tea, coffee, bread, etc., were not considered as TFBs, unless there was a distinguishable variety (e.g., cappuccino coffee in Italy). ^*^Nonetheless, some consumers mentioned an ingredient or basic product, but they meant a specific dish/snack/drink. If applicable, the food/drink was considered as traditional and later on classified in its respective group.
5. It can be present in other countries, and recognised as traditional from another country	Many traditional foods are present in several countries. Nevertheless, still perceived as TFBs by many of them (usually different varieties exist).

* *Exemptions apply*.

The criteria were developed by the authors following a deductive approach. However, special exemptions were inductively included during the data analysis. The first criterion “*Is representative of a country's cuisine”* was the foremost inclusion criteria developed since it reflected the definition of TFBs used in this study. The remaining criteria were created to ensure that the foods and beverages mentioned by the respondents were in line with the definition and the instructions given in the research instrument. Furthermore, the third and fourth criteria were added because this study did not aim to learn about ingredients, but on the national cuisine in terms of dishes, snacks, and beverages that have some form of processing. Finally, criterion six “*It can be present in other countries, and recognised as traditional from another country*” despite the fact that TFBs can coexist in different countries, it is usually with some level of variation, but most importantly, it was included because the study was focused on the perceptions of inhabitants. Special exemptions were developed and validated during the data analysis together with the natives from each country. For instance, criterion 2 was flexible in Indonesia due to the large number of islands. If dishes and beverages mentioned by respondents from different islands were comparable—in terms of ingredients, shape, and type of food/beverage—they were grouped into the same category to avoid over representing similar TFBs. The second exemption was in criterion 4, since a few respondents mentioned an ingredient or basic product, but they meant a specific dish/snack/drink. If applicable, the food/drink was considered as traditional and later on classified in its respective group.

After applying the five criteria, data were excluded under three conditions: (i) when respondents listed more than three non-traditional foods and beverages (TFBs), it was assumed that they did not understand the question; (ii) when respondents did not name at least one of the TFBs mentioned by 13–20% of the sample of the country; and (iii) since only two respondents were in the age cohort 50+ in Brazil and one respondent in Indonesia (three in total), this cohort was eliminated from the analysis in these countries to avoid over-representing an age cohort with a very low sample size. Under conditions (i) and (ii) the answers provided by 293 respondents across countries were eliminated, representing 14% of the pooled sample.

### Thematic Analysis

After preprocessing the data, a systematic qualitative analysis of the TFBs mentioned was carried out through thematic analysis by coding and classifying the data to determine the most frequently mentioned TFBs per age cohort (21). First, the spelling of TFBs was standardised by the natives from each country to unify the answers into one code per TFBs. Afterwards, the foods and beverages were classified into categories when respondents mentioned varieties of a product. The TFBs mentioned by at least 13–20% of respondents in each country, from the largest cities and other cities, were further analysed. To determine the threshold, we focused on the representativity of the diversity of TFBs at a national level by considering the answers given by respondents from the largest cities and other cities of each country. Two to three natives from each country were asked to act as experts with the aim of crosschecking whether the TFBs selected at different proportions (50–20%) were representative of the cuisine of the country, that is, that they could be classified as TFBs based on the definition used in this study and if they were available nationwide. In most of the countries, a threshold of 20% captured what the study aimed for. However, in China and Indonesia, this threshold was capturing fewer TFBs (five approximately). Since these countries are either large or spread into several islands, which implies a larger variation, we decided to extend the list of TFBs to a minimum of 10 to be able to look for similarities and differences in the perceptions of the four age cohorts. Hence, in China and Indonesia, a threshold of 13% was used to select the TFBs to be further analysed.

In this study, we aimed at having true representations of the traditional nature of the TFBs selected. Therefore, the figures displayed show the TFBs in their original language when no English translation was available. Nonetheless, Supplementary Table 2 shows a list of the TFBs with an English translation or short description, which were validated by two natives from each country.

### Statistical Analyses

Since this study is explorative by nature, data was mainly analysed following a qualitative approach. Nonetheless, the outcomes were complemented from a quantitative perspective to have a better understanding of the perceptions of our sample. Correspondence analyses (CA) of column profiles aiming to identify the main relationships between the TFBs mentioned and different age cohorts on a two-dimensional perception map (22–24) were carried out using the FactoMineR and factoextra packages in R Software (version 3.5.0) Hierarchical clustering on principle components was performed to determine clusters between the age cohorts studied by using the FactoMineR package. Euclidean distances were calculated and the trees were built using the Ward method The relationship between age cohorts, gender, and place of residence (predictor variables) and respondents mentioning a specific TFBs (binary dependent variable) was determined by binary logistic regression in a stepwise approach (PIN = 0.05, POUT = 0.10) where the cut-off value for classification was set to 0.5. Bonferroni–Holm correction was applied to control the family-wise rate due to the multiple hypotheses tested per country (25). These analyses were performed in SPSS version 25.

## Results

### Socio-Demographics of the Sample

We summarised the socio-demographic aspects of the pooled sample and per country in Table 2. The pooled sample consisted of 1,784 respondents, where 65% were women and 35% men. The mean age was 36 years and the best-represented age cohorts were C1 (18–29 years) and C2 (30–39 years). Most respondents in each country lived in other cities, i.e., different than the largest city, except for Brazilians and Chileans. Regarding their occupation, most of them were workers employed with salary (60%), followed by students (16%), and student workers (7%).

**Table 2:** Sample demographics by pooled sample and by country.

	**America**				**Europe**			**Asia**		
	**Pooled sample**	**Mexico**	**Brazil**	**Chile**	**The Netherlands**	**Italy**	**Hungary**	**China**	**Japan**	**Indonesia**
**Sample size (** ***n*** **=)**	1,784	119	77	891	122	159	41	125	78	172
**Gender (%)**										
Female	65	75	94	60	70	72	83	58	38	74
Male	35	25	6	40	30	28	17	42	62	26
**Age (%)**										
Mean	36	35	33	38	39	40	43	30	33	31
Range	18–83	18–66	23–49	18–73	20–72	21–79	27–63	18–83	18–74	19–49
C1 (18-29)	33	30	29	23	35	33	12	80	50	52
C2 (30-39)	35	45	56	41	28	17	32	7	27	37
C3 (40-49)	18	14	16	23	9	21	22	•	18	10
C4 (50 +)	12	10	•	13	28	28	34	•	5	•
C5 (40 +)	1	•	•	•	•	•	•	13	•	•
**Place of residence (%)**										
Largest city	55	35	60	78	33	31	34	12	10	46
Other cities	45	65	40	22	67	69	66	88	90	54
**Occupation (%)**										
Student	16	10	10	10	15	13	•	54	24	26
Student worker	7	8	18	7	5	6	•	1	8	6
Worker in an employ with salary	60	58	30	70	61	55	85	29	63	41
Freelance worker	6	11	29	^*^	7	16	2	6	3	11
Houseman/housewife	5	9	3	5	2	5	2	4	3	14
Unemployed/volunteering	5	2	10	7	3	3	2	2	•	2
Retired	2	2	•	2	6	3	7	4	•	•

* *This alternative was not present in this survey*.

• *No respondents from these groups*.

### Similarities and Differences Among Countries, Age Cohorts, Gender, and Place of Residence

**Figure 1:**
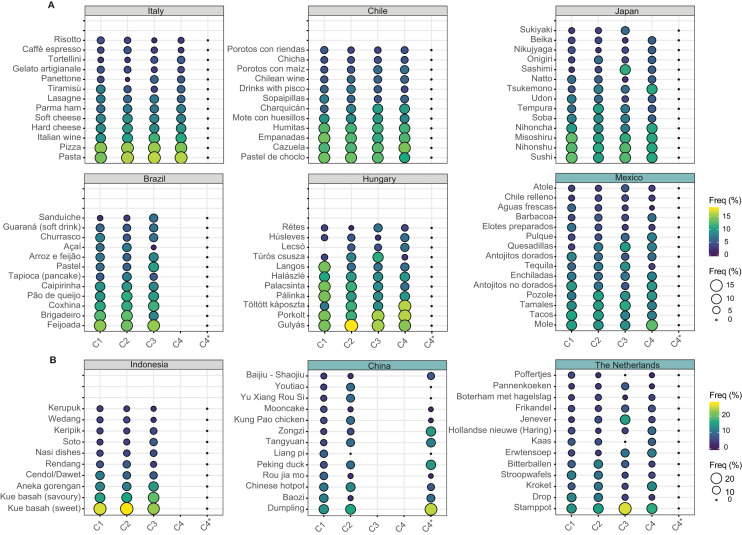
Percentage frequency of traditional foods and beverages (mentioned by ≥ 13% respondents) relative to each age cohort by country. Countries with grey background represent those having a homogeneous food identity and the countries with a blue background those with a heterogeneous food identity. **(A,B)** Represent different plotting scales. C1 = 18–29 years of age, C2 = 30–39 years of age, C3 = 40–49 years of age, C4 = 50+ years of age; and C4^*^ = 40+ years of age.

Based on the results, it was possible to distinguish two sets of countries, those having a homogeneous food identity, i.e., with a distinctive core of TFBs highly mentioned across age cohorts, and those with a heterogeneous food identity, i.e., a wide variety of TFBs mentioned in similar proportions (see Figure 1). Three countries were classified in the second group (Mexico, China, and The Netherlands) whereas the remaining countries showed a homogeneous food identity.

**Figure 2:**
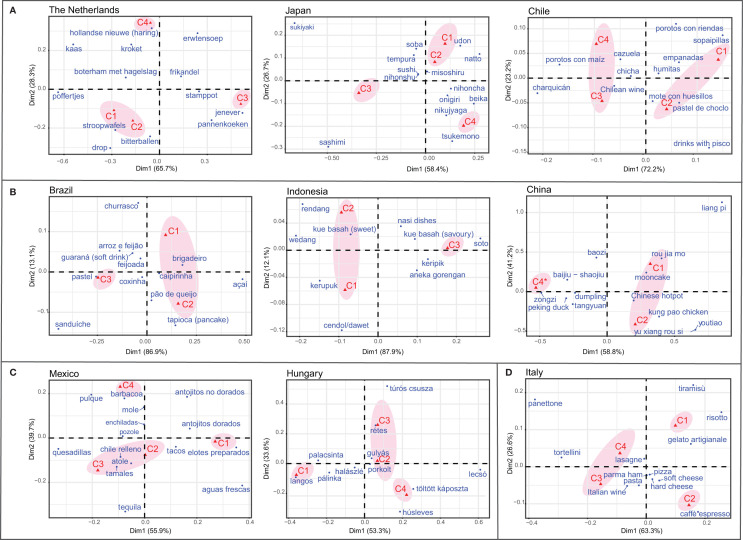
Perceptual maps of the correspondence analyses representing the relationships between the traditional foods and beverages mentioned and the different age cohorts by country. **(A)** groups the countries where age cohorts C1 and C2 were clustered together; **(B)** groups the countries where age cohorts C1–C3 or C1-C2-C4* (China) were analysed and C1 and C2 were clustered together; **(C)** groups countries where middle-age cohorts C2 and C3 were clustered together; and **(D)** shows the Italian clusters where oldest age cohorts C3 and C4 were in the same cluster. C1 = 18–29 years of age, C2 = 30–39 years of age, C3 = 40–49 years of age, C4 = 50+ years of age; and C4* = 40+ years of age. Clusters ovals were added to the perceptual maps using adobe illustrator to aid with the interpretation.

Similarities and differences in the TFBs mentioned by the age cohorts within and between countries were identified after both quantitative and qualitative analyses. In most of the countries, the youngest age cohorts C1 and C2 (18–29 and 30–39) were clustered together, for example, Japan, China, Indonesia, Chile, Brazil, and The Netherlands showing a high similarity in the foods and beverages perceived as traditional (Figure 2). On the other hand, middle-aged cohorts (30–39 and 40–49) shared similar TFBs in Mexico and Hungary. Surprisingly, in Italy, the distribution was the opposite. The oldest age cohorts (40–49 and 50+) were grouped together and differed from the youngest.

Concerning the typology of TFBs mentioned by each age cohort, a distinctive pattern was spotted. In several countries (The Netherlands, Chile, Mexico, Hungary, and Italy) respondents of youngest age cohorts more often mentioned TFBs belonging to snacks, food products, or beverages consumed on-the-go and/or easy-to-prepare. Interestingly, some TFBs were only mentioned by youngest age cohorts, for example, “açaí” in Brazil. On the contrary, older age cohorts (40–49, 40+, and 50+) were more inclined towards mentioning foods that require long preparation times and are related to home consumption. Another interesting example comes from Japan, where “sashimi”, a food with short preparation time, was more mentioned by oldest age cohorts (30–49, and 50+) suggesting a decline in the perception of this food as traditional in younger generations.

The binary logistic regression analyses identified significant differences in the probability of mentioning a specific TFBs mentioned across age cohorts – after Bonferroni–Holm correction – in Chile and The Netherlands using the oldest age cohorts (40+ or 50+) as a reference group (Supplementary Table 1A). For instance, in The Netherlands respondents between 18 and 39 years old (C1 and C2) were more likely to mention snacks (“drop,” “stroopwafels,” and “bitterballen”) as a TFBs from The Netherlands when compared with the reference group (50+). Similarly, in Chile, the snack “sopaipillas” was more probably mentioned by respondents between 18 and 39 years old (C1/C2). On the contrary, the savoury dish “charquicán” was less likely to be mentioned by youngest age cohorts when compared to C4.

Regarding gender, significant differences—after Bonferroni–Holm correction—between women and men were only found in Chile. Nonetheless, similar trends were also detected in other countries (Supplementary Table 1B). Even though respondents were asked to mention TFBs available nationwide, we identified significant differences—after Bonferroni–Holm correction—in two distilled beverages/cocktails from Chile and Brazil between respondents living in the largest city when compared with the rest of the country (Supplementary Table 1C). “Drinks with pisco” from Chile and “Caipirinha” from Brazil were 1.7 and 4.8 times more likely to be mentioned by respondents living in the largest city when compared with the other cities, respectively.

### Type of TFBs by Country and Consumption Moment

**Figure 3:**
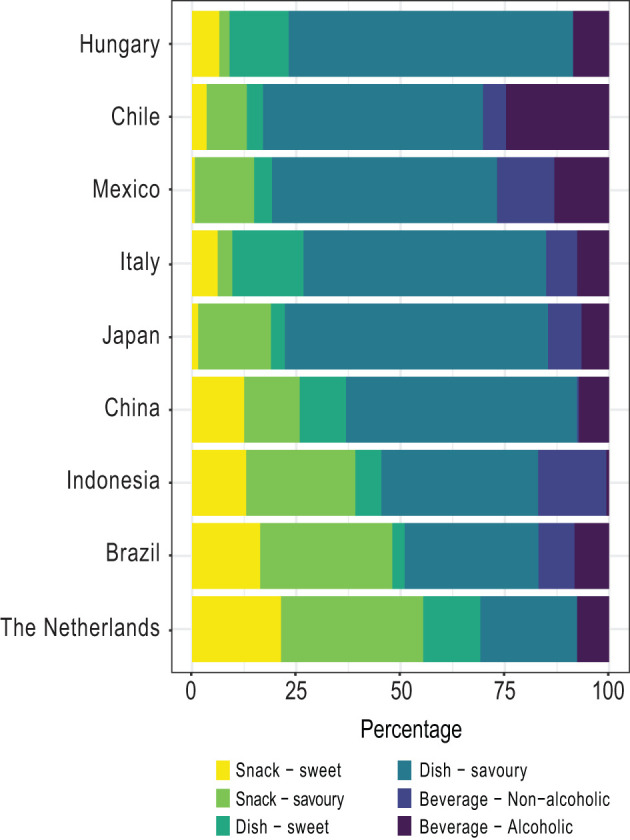
Type of traditional foods and beverages by country. Percentage of type of foods/beverages relative to the number of traditional foods and beverages most frequently mentioned by country (stated by ≥ 13% of respondents).

**Figure 4:**
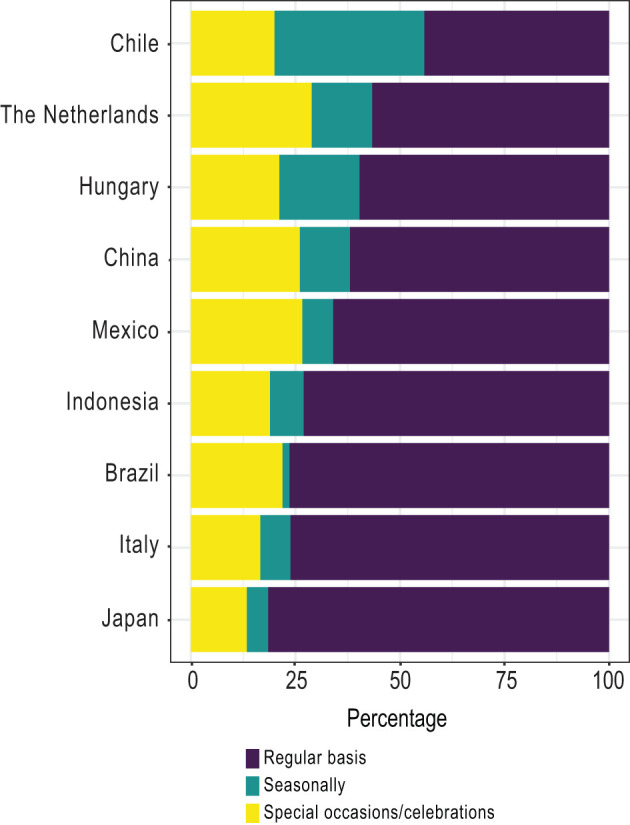
Consumption moment of traditional foods/beverages by country. Percentage of consumption moment situations of TFBs relative to the number of traditional foods/beverages most frequently mentioned by country (stated by ≥ 13% of respondents).

The distribution of the type of TFBs most frequently mentioned was examined by considering three large categories namely dishes, snacks, and beverages, which were subcategorised into savoury and/or sweet foods and (non) alcoholic beverages, respectively. A majority of the countries (6/9) mainly mentioned dishes followed by snacks and beverages (Figure 3), but the opposite happened in the Netherlands, Brazil, and Indonesia, which mentioned more snacks than dishes. Regarding the beverages, the Latin American countries (Chile, Mexico, and Brazil) stated a larger proportion of them when compared with the other countries. Concerning the subcategories, between 5 and 20% of traditional foods were sweet across the countries and mainly alcoholic beverages were mentioned. In most of the countries, TFBs were primarily consumed on a regular basis followed by special occasions and celebrations (Figure 4). However, in Chile the distribution changed since nearly 45% were consumed on a regular basis, followed by seasonal consumption (35%).

## Discussion

Traditional foods and beverages represent the food identity and continuity of culinary traditions across generations in a country. In addition, they reflect the dietary habits of the inhabitants of a country since TFBs have been consumed for a long period of time (2). Nonetheless, it is unknown if the perception of consumers towards TFBs is changing over time and how it is affecting different cultures. Learning about their perceptions can help to have a better understanding of how traditions and food identity are influenced by globalisation and new lifestyles across the world. In this context, our research is helping to fill in that gap by analysing the differences in food identity by means of the TFBs currently consumed in nine countries with different cultural backgrounds. The core findings of our study are discussed below.

### Homogeneous vs. Heterogeneous Food and Beverage Identity

Most countries showed a homogeneous food identity, meaning that they have a distinctive core of TFBs highly mentioned across age cohorts. However large countries with strong regional cuisines—such as Mexico and China—were classified as having a heterogeneous food identity. Hereof, throughout the years, countries have been impacted by different phenomena that have altered their food habits (26). This alteration varies depending on their exposure to other cultures and their diets (27, 28), but also based on its size, administrative divisions, and interconnectivity, which could explain the heterogeneity obtained in these two countries. Interestingly, The Netherlands also followed a similar pattern, which could be related to its high globalisation and immigration levels (29, 30).

### Younger vs. Older Generations

During lifespan, we are exposed to several TFBs, which set the learned preferences of the consumers in terms of ingredients, textures, flavour profiles, cooking skills, among others (31). Nonetheless, these preferences are believed to change in function of age of the consumer, his/her lifestyles, country, and current societal trends, which may explain why our results showed differences in the perceptions of TFBs between generations. Overall, youngest age cohorts (C1–C2) shared similar perceptions regarding the foods and beverages considered as traditional whereas older age cohorts (C3–C4) were usually dissimilar. Differences were also spotted between the types of TFBs since convenience foods that are usually consumed as snacks on-the-go were most probably mentioned by the youngest age cohorts. On the contrary, older age cohorts were more inclined towards time-consuming preparations or specific TFBs. In this respect, previous studies have obtained similar results when attempting to unravel the key predictors driving convenience in food consumption (32) and the consumer segments that are more inclined towards convenience food products (33). The former identified age (young participants), naturalness, and nutrition knowledge as the main drivers among different types of processed foods, and low cooking skills were linked to the consumption of moderately to highly processed foods. The latter research reported that consumers seeking convenience and those with fewer cooking skills were prone to purchase convenience foods. Our results suggest that the convenience trend is also impacting the habits of young generations when consuming TFBs.

Notably, special trends were spotted regarding the introduction of new TFBs, or new versions of preexisting TFBs, lead by the youngest age cohorts. For instance, “tiramisu,” a sweet dessert—not present in kitchen books edited before 1960 (34)—was mainly mentioned by C1–C2, opposite to “panettone,” a sweet cake commonly consumed during Christmas, which was primarily stated by older age cohorts. Another interesting case is “açaí” in Brazil, which nowadays is highly consumed as “açaí na tigela” (bowl with açaí paste topped with fruit slices), which was spread in the market, both nationally and internationally, in recent years following the healthy trend because of its high antioxidant capacity and anthocyanin content (35, 36). Similarly, “bitterballen” a crunchy deep-fried ball filled with meat ragout was most frequently mentioned by younger age cohorts, while “kroket”—a roll with the same ingredients and taste as “bitterballen,” but with a different consumption moment—was mostly mentioned by C3. These results suggest that new societal trends may have an impact on the food identity of countries lead by younger generations.

### Type of TFBs by Country and Consumption Moment

It is well-known that different dietary habits exist across the world, including a wide diversity in the type and amount of foods and beverages consumed during the day (37–39). Regarding the type of TFBs and its consumption moment, our results showed that overall the TFBs most frequently mentioned were savoury dishes consumed on a regular basis during breakfast, lunch, and/or dinner. These outcomes reflect the importance of consuming TFBs in the main meals throughout the day in most countries. However, a different pattern was identified in Brazil, The Netherlands, and Indonesia, where snacks lead the list. This finding might suggest that snack-eating habits are rooted in the traditional cuisine in a country-dependent manner. Interestingly, Chilean respondents stated a large seasonal consumption (35%) of TFBs, which may be linked to the seasonal produce and low off-season imports of fresh commodities (40).

Traditional foods and beverages represent the core of the cultural identity of a country (2). TFBs also play an important societal role at family and regional level (11). The knowledge gathered in this study identified some common patterns occurring in different countries worldwide. The perception of food and beverage products being considered as traditional varies across age cohorts, suggesting a shift of younger generations towards convenience TFBs. This variation is less in countries with a homogeneous food identity. Current societal trends in food consumption, for example, convenience and healthiness, are impacting the food culture and identity of countries, and societal trends are expected to play an important role in the further diversification of the TFBs perceived and consumed.

## Limitations

The respondents who participated in the survey were likely those interested in the topic of (traditional) foods, which explains the observed higher response rate of women because in many countries they are mainly responsible for cooking foods at home (41). In addition, the convenience sampling method using social media led to an underrepresentation of the older age cohorts in many countries, which eliminates the possibility to meaningfully weigh data to adjust for the disproportional representation of the number of people within each age cohort. In sum, our exploratory analyses discovered differences between the younger and older age cohorts at a qualitative level, but we have insufficient means to determine the prevalence of food perceptions of the consumer. Future research should shed light on quantitative issues by realising larger samples stratified over gender and age cohorts.

## Data Availability Statement

The data are not publicly available due to them containing information that could compromise research participant privacy. The dataset that support the findings of this study are available upon reasonable request from the corresponding author Lucía Frez-Muñoz, lucia.frezmunoz@wur.nl.

## Ethics Statement

The studies involving human participants were reviewed and approved by Social Sciences Ethics Committee at Wageningen University Research. The participants provided their implied informed consent to participate in this study.

## Author Contributions

LF-M, VF, and BS designed the study and participated in data collection. LF-M analysed the data and produced the figures. JK guided the statistical analyses. With the obtained data, main decisions concerning the analyses, and the final article were jointly discussed by all authors.

## Conflict of Interest

The authors declare that the research was conducted in the absence of any commercial or financial relationships that could be construed as a potential conflict of interest.

## Publisher's Note

All claims expressed in this article are solely those of the authors and do not necessarily represent those of their affiliated organizations, or those of the publisher, the editors and the reviewers. Any product that may be evaluated in this article, or claim that may be made by its manufacturer, is not guaranteed or endorsed by the publisher.
