# Supplementary material for: The Food Identity of Countries Differs Between Younger and Older Generations: A Cross-Sectional Study in American, European and Asian Countries
Source: Front Nutr. 2021 Aug 16;8:653039. doi: 10.3389/fnut.2021.653039 (PMC8415227; doi:10.3389/fnut.2021.653039)
Supplement: Supplementary file 1 [file Data_Sheet_1.docx]

**Appendix:** Supplementary material

**Table captions**

**Supplementary Table S1.a** Binary logistic regression results by age cohort and country

**Supplementary Table S1.b** Binary logistic regression results by gender and country

**Supplementary Table S1.c** Binary logistic regression results by place of residence and country

**Supplementary Table S2.** List of TFB in their native name and short description

**Table S1.a** Binary logistic regression results by age cohort and country

| Country | TFB | **Model** | | | Nagelkerke R Square | **Hosmer and Lemeshow Test** | | | Classification  table (%) | **Variables in the Equation** | | | | | | | | |
| --- | --- | --- | --- | --- | --- | --- | --- | --- | --- | --- | --- | --- | --- | --- | --- | --- | --- | --- |
|  |  | Chi-Square | *df* | Sig* |  | Chi-Square | *df* | Sig |  |  | B | S.E. | Wald | *df* | Sig. | Exp(B) | 95% C.I.for EXP(B) | |
|  |  |  |  |  |  |  |  |  |  |  |  |  |  |  |  |  | Lower | Upper |
| **Chile** |  |  |  |  |  |  |  |  |  |  |  |  |  |  |  |  |  |  |
|  | Charquicán | 25.910 | 3 | 0.000 | 0.038 | 0.000 | 2 | 1.000 | 58.1 | Age cohorts |  |  | 24.922 | 3 | 0.000 |  |  |  |
|  |  |  |  |  |  |  |  |  |  | C1 (18-29) | -0.839 | 0.242 | 12.017 | 1 | 0.001 | 0.432 | 0.269 | 0.694 |
|  |  |  |  |  |  |  |  |  |  | C2 (30-39) | -0.282 | 0.216 | 1.704 | 1 | 0.192 | 0.755 | 0.494 | 1.152 |
|  |  |  |  |  |  |  |  |  |  | C3 (40-49) | 0.145 | 0.234 | 0.386 | 1 | 0.534 | 1.156 | 0.731 | 1.829 |
|  |  |  |  |  |  |  |  |  |  | Constant | 0.018 | 0.188 | 0.009 | 1 | 0.925 | 1.018 |  |  |
|  | Sopaipillas | 13.371 | 3 | 0.004 | 0.021 | 0.000 | 2 | 1.000 | 68.4 | Age cohorts |  |  | 12.667 | 3 | 0.005 |  |  |  |
|  |  |  |  |  |  |  |  |  |  | C1 (18-29) | 0.589 | 0.278 | 4.477 | 1 | 0.034 | 1.802 | 1.044 | 3.108 |
|  |  |  |  |  |  |  |  |  |  | C2 (30-39) | 0.839 | 0.257 | 10.616 | 1 | 0.001 | 2.314 | 1.397 | 3.833 |
|  |  |  |  |  |  |  |  |  |  | C3 (40-49) | 0.431 | 0.280 | 2.378 | 1 | 0.123 | 1.539 | 0.890 | 2.663 |
|  |  |  |  |  |  |  |  |  |  | Constant | -1.364 | 0.234 | 34.097 | 1 | 0.000 | 0.256 |  |  |
|  | Pastel de choclo | 12.494 | 3 | NS |  |  |  |  |  |  |  |  |  |  |  |  |  |  |
|  | Empanadas | 11.274 | 3 | NS |  |  |  |  |  |  |  |  |  |  |  |  |  |  |
|  | Drinks with pisco | 12.648 | 3 | NS |  |  |  |  |  |  |  |  |  |  |  |  |  |  |
|  | Porotos con maiz | 8.421 | 3 | NS |  |  |  |  |  |  |  |  |  |  |  |  |  |  |
| **The Netherlands** | |  |  |  |  |  |  |  |  |  |  |  |  |  |  |  |  |  |
|  | Drop | 29.953 | 3 | 0.000 | 0.290 | 0.000 | 2.000 | 1.000 | 73 | Age cohorts |  |  | 24.718 | 3 | 0.000 |  |  |  |
|  |  |  |  |  |  |  |  |  |  | C1 (18-29) | 2.418 | 0.550 | 19.353 | 1 | 0.000 | 11.221 | 3.821 | 32.949 |
|  |  |  |  |  |  |  |  |  |  | C2 (30-39) | 1.829 | 0.552 | 10.994 | 1 | 0.001 | 6.231 | 2.113 | 18.374 |
|  |  |  |  |  |  |  |  |  |  | C3 (40-49) | -0.154 | 0.889 | 0.030 | 1 | 0.862 | 0.857 | 0.150 | 4.899 |
|  |  |  |  |  |  |  |  |  |  | Constant | -1.350 | 0.424 | 10.130 | 1 | 0.001 | 0.259 |  |  |
|  | Stroopwafels | 19.029 | 3 | 0.000 | 0.193 | 0.000 | 2.000 | 1.000 | 68 | Age cohorts |  |  | 16.651 | 3 | 0.001 |  |  |  |
|  |  |  |  |  |  |  |  |  |  | C1 (18-29) | 1.750 | 0.507 | 11.914 | 1 | 0.001 | 5.754 | 2.130 | 15.542 |
|  |  |  |  |  |  |  |  |  |  | C2 (30-39) | 1.378 | 0.522 | 6.971 | 1 | 0.008 | 3.968 | 1.426 | 11.040 |
|  |  |  |  |  |  |  |  |  |  | C3 (40-49) | -0.482 | 0.873 | 0.305 | 1 | 0.581 | 0.617 | 0.112 | 3.417 |
|  |  |  |  |  |  |  |  |  |  | Constant | -1.022 | 0.389 | 6.907 | 1 | 0.009 | 0.360 |  |  |
|  | Bitterballen | 13.482 | 3 | 0.004 | 0.140 | 0.000 | 2.000 | 1.000 | 64.8 | Age cohorts |  |  | 12.251 | 3 | 0.007 |  |  |  |
|  |  |  |  |  |  |  |  |  |  | C1 (18-29) | 1.039 | 0.507 | 4.201 | 1 | 0.040 | 2.826 | 1.046 | 7.632 |
|  |  |  |  |  |  |  |  |  |  | C2 (30-39) | 1.785 | 0.541 | 10.900 | 1 | 0.001 | 5.958 | 2.065 | 17.190 |
|  |  |  |  |  |  |  |  |  |  | C3 (40-49) | 0.198 | 0.789 | 0.063 | 1 | 0.802 | 1.219 | 0.260 | 5.716 |
|  |  |  |  |  |  |  |  |  |  | Constant | -1.179 | 0.404 | 8.499 | 1 | 0.004 | 0.308 |  |  |
|  | Kroket | 8.927 | 3 | NS |  |  |  |  |  |  |  |  |  |  |  |  |  |  |
| **Japan** |  |  |  |  |  |  |  |  |  |  |  |  |  |  |  |  |  |  |
|  | Sashimi | 13.134 | 3 | NS |  |  |  |  |  |  |  |  |  |  |  |  |  |  |
| **Brazil** |  |  |  |  |  |  |  |  |  |  |  |  |  |  |  |  |  |  |
|  | Açaí | 6.191 | 2 | NS |  |  |  |  |  |  |  |  |  |  |  |  |  |  |
| **Mexico** |  |  |  |  |  |  |  |  |  |  |  |  |  |  |  |  |  |  |
|  | Quesadillas | 9.357 | 3 | NS |  |  |  |  |  |  |  |  |  |  |  |  |  |  |
| **Italy** |  |  |  |  |  |  |  |  |  |  |  |  |  |  |  |  |  |  |
|  | Tiramisu | 8.187 | 3 | NS |  |  |  |  |  |  |  |  |  |  |  |  |  |  |
| **China** |  |  |  |  |  |  |  |  |  |  |  |  |  |  |  |  |  |  |
|  | Peking duck | 6.275 | 2 | NS |  |  |  |  |  |  |  |  |  |  |  |  |  |  |
| **Indonesia** | n/a |  |  |  |  |  |  |  |  |  |  |  |  |  |  |  |  |  |
| **Hungary** | n/a |  |  |  |  |  |  |  |  |  |  |  |  |  |  |  |  |  |

***** : p-value of the chi-square statistic obtained in the binary logistic regression model subject to Bonferroni-Holm correction

**NS**.: non-significant result after correcting for family wise-error by using Bonferroni-Holm correction

**n/a**.: Not applicable, i.e. equal probability of mentioning the TFBs from that country across age cohorts

**Table S1.b** Binary logistic regression results by gender and country

| Country | TFB | **Model** | | | Nagelkerke R Square | **Hosmer and Lemeshow Test** | | | Classification table (%) | **Variables in the Equation** | | | | | | | | |
| --- | --- | --- | --- | --- | --- | --- | --- | --- | --- | --- | --- | --- | --- | --- | --- | --- | --- | --- |
|  |  | Chi-Square | *df* | Sig* |  | Chi-Square | *df* | Sig |  |  | B | S.E. | Wald | *df* | Sig. | Exp(B) | 95% C.I.for EXP(B) | |
|  |  |  |  |  |  |  |  |  |  |  |  |  |  |  |  |  | Lower | Upper |
| **Chile** |  |  |  |  |  |  |  |  |  |  |  |  |  |  |  |  |  |  |
|  | Porotos con maiz | 11.426 | 1 | 0.001 | 0.018 | 0.000 | 0 |  | 70.7 | Female | 0.519 | 0.156 | 11.106 | 1 | 0.001 | 1.680 | 1.238 | 2.280 |
|  |  |  |  |  |  |  |  |  |  | Constant | -1.206 | 0.126 | 91.855 | 1 | 0.000 | 0.299 |  |  |
|  | Humitas | 5.629 | 1 | NS |  |  |  |  |  |  |  |  |  |  |  |  |  |  |
| **Brazil** |  |  |  |  |  |  |  |  |  |  |  |  |  |  |  |  |  |  |
|  | Guaraná (soft drink) | 4.843 | 1 | NS |  |  |  |  |  |  |  |  |  |  |  |  |  |  |
| **Mexico** |  |  |  |  |  |  |  |  |  |  |  |  |  |  |  |  |  |  |
|  | Mole | 6.275 | 1 | NS |  |  |  |  |  |  |  |  |  |  |  |  |  |  |
| **Italy** |  |  |  |  |  |  |  |  |  |  |  |  |  |  |  |  |  |  |
|  | Pizza | 5.633 | 1 | NS |  |  |  |  |  |  |  |  |  |  |  |  |  |  |
| **The Netherlands** | |  |  |  |  |  |  |  |  |  |  |  |  |  |  |  |  |  |
|  | Frikandel | 5.944 | 1 | NS. |  |  |  |  |  |  |  |  |  |  |  |  |  |  |
| **Indonesia** |  |  |  |  |  |  |  |  |  |  |  |  |  |  |  |  |  |  |
|  | Kue basah (sweet) | 4.641 | 1 | NS |  |  |  |  |  |  |  |  |  |  |  |  |  |  |
| **Hungary** | n/a |  |  |  |  |  |  |  |  |  |  |  |  |  |  |  |  |  |
| **China** | n/a |  |  |  |  |  |  |  |  |  |  |  |  |  |  |  |  |  |
| **Japan** | n/a |  |  |  |  |  |  |  |  |  |  |  |  |  |  |  |  |  |

***** : p-value of the chi-square statistic obtained in the binary logistic regression model subject to Bonferroni-Holm correction

**NS**.: non-significant result after correcting for family wise-error by using Bonferroni-Holm correction

**n/a**.: Not applicable, i.e. equal probability of mentioning the TFBs from that country between gender

**Table S1.b** Binary logistic regression results by place of residence and country

| Country | TFB | **Model** | | | Nagelkerke R Square | **Hosmer and Lemeshow Test** | | | Classification table (%) | **Variables in the Equation** | | | | | | | | |
| --- | --- | --- | --- | --- | --- | --- | --- | --- | --- | --- | --- | --- | --- | --- | --- | --- | --- | --- |
|  |  | Chi-Square | *df* | Sig* |  | Chi-Square | *df* | Sig |  |  | B | S.E. | Wald | *df* | Sig. | Exp(B) | 95% C.I.for EXP(B) | |
|  |  |  |  |  |  |  |  |  |  |  |  |  |  |  |  |  | Lower | Upper |
| **Chile** |  |  |  |  |  |  |  |  |  |  |  |  |  |  |  |  |  |  |
|  | Drinks with pisco | 8.996 | 1 | 0.003 | 0.014 | 0.000 | 0 |  | 68.4 | Largest city | 0.543 | 0.186 | 8.500 | 1 | 0.004 | 1.721 | 1.195 | 2.480 |
|  |  |  |  |  |  |  |  |  |  | Constant | -1.202 | 0.168 | 51.081 | 1 | 0.000 | 0.301 |  |  |
|  | Charquican | 8.006 | 1 | NS |  |  |  |  |  |  |  |  |  |  |  |  |  |  |
|  | Porotos con maiz | 7.632 | 1 | NS |  |  |  |  |  |  |  |  |  |  |  |  |  |  |
| **Brazil** |  |  |  |  |  |  |  |  |  |  |  |  |  |  |  |  |  |  |
|  | Caipirinha | 10.588 | 1 | 0.001 | 0.172 | 0.000 | 0 |  | 68.8 | Largest city | 1.569 | 0.500 | 9.831 | 1 | 0.002 | 4.800 | 1.800 | 12.797 |
|  |  |  |  |  |  |  |  |  |  | Constant | -0.742 | 0.384 | 3.729 | 1 | 0.053 | 0.476 |  |  |
|  | Coxinha | 6.219 | 1 | NS |  |  |  |  |  |  |  |  |  |  |  |  |  |  |
|  | Churrasco | 6.219 | 1 | NS |  |  |  |  |  |  |  |  |  |  |  |  |  |  |
| **The Netherlands** | |  |  |  |  |  |  |  |  |  |  |  |  |  |  |  |  |  |
|  | Hollandse nieuwe (Haring) | 3.943 | 1 | NS |  |  |  |  |  |  |  |  |  |  |  |  |  |  |
| **Mexico** |  |  |  |  |  |  |  |  |  |  |  |  |  |  |  |  |  |  |
|  | Elotes preparados | 4.039 | 1 | NS |  |  |  |  |  |  |  |  |  |  |  |  |  |  |
| **Japan** |  |  |  |  |  |  |  |  |  |  |  |  |  |  |  |  |  |  |
|  | Beika | 3.929 | 1 | NS |  |  |  |  |  |  |  |  |  |  |  |  |  |  |
| **Indonesia** |  |  |  |  |  |  |  |  |  |  |  |  |  |  |  |  |  |  |
|  | Aneka Gorengan | 4.375 | 1 | NS |  |  |  |  |  |  |  |  |  |  |  |  |  |  |
|  | Nasi | 5.653 | 1 | NS |  |  |  |  |  |  |  |  |  |  |  |  |  |  |
|  | Kerupuk | 6.041 | 1 | NS |  |  |  |  |  |  |  |  |  |  |  |  |  |  |
| **China** |  |  |  |  |  |  |  |  |  |  |  |  |  |  |  |  |  |  |
|  | Rou jia mo | 4.034 | 1 | NS |  |  |  |  |  |  |  |  |  |  |  |  |  |  |
|  | Peking duck | 6.069 | 1 | NS |  |  |  |  |  |  |  |  |  |  |  |  |  |  |
| **Italy** | n/a |  |  |  |  |  |  |  |  |  |  |  |  |  |  |  |  |  |
| **Hungary** | n/a |  |  |  |  |  |  |  |  |  |  |  |  |  |  |  |  |  |

***** : p-value of the chi-square statistic obtained in the binary logistic regression model subject to Bonferroni-Holm correction

**NS**.: non-significant result after correcting for family wise-error by using Bonferroni-Holm correction

**n/a**.: Not applicable, i.e. equal probability of mentioning the TFBs from that country between place of residence

**Table S2** List of TFB in their native name and short description

| Country | TFB native name or as in figure 2 | Translated name/short description |
| --- | --- | --- |
| The Netherlands |  |  |
|  | Poffertjes | Small round pancakes |
|  | Pannenkoeken | Pancakes |
|  | Boterham met hagelslag | Sliced bread topped with chocolate sprinkles |
|  | Frikandel | Brown sausage |
|  | Jenever | Jenever |
|  | Hollandse nieuwe (haring) | Herring with onion |
|  | Kaas | Cheese |
|  | Erwtensoep | Pea soup |
|  | Bitterballen | Crunchy deep-fried ball filled with meat ragout |
|  | Stroopwafels | Wafers with caramel syrup |
|  | Kroket | Crunchy deep-fried roll filled with meat ragout |
|  | Drop | Liquorice |
|  | Stamppot | Mashed potatoes with vegetables |
| Italy |  |  |
|  | Risotto | Creamy rice |
|  | Caffè espresso | Espresso coffee |
|  | Tortellini | Filled ring-shaped pasta |
|  | Gelato artigianale | Artisanal ice cream |
|  | Panettone | Christmas sweet bread-like cake |
|  | Tiramisù | Tiramisu |
|  | Lasagna | Layered pasta |
|  | Parma ham | Parma ham |
|  | Soft cheese | Soft cheese |
|  | Hard cheese | Hard cheese |
|  | Italian wine | Italian wine |
|  | Pizza | Pizza |
|  | Pasta | Pasta dishes |
| Hungary |  |  |
|  | Rétes | Strudel |
|  | Lecsó | Tomato-pepper stew |
|  | Húsleves | Meat soup |
|  | Túrós csusza | Noodles with quark |
|  | Lángos | Savoury fried bread |
|  | Palacsinta | Pancakes |
|  | Halászlé | Fisherman's soup (paprika-tomato) |
|  | Pálinka | Fruity brandy |
|  | Töltött káposzta | Filled cabbage leaves |
|  | Porkolt | Stew with beef and onion |
|  | Gulyás | Goulash |
| Brazil |  |  |
|  | Sanduíche | Brazilian sandwich |
|  | Guaraná | Guaraná soft drink |
|  | Churrasco | Barbeque |
|  | Açaí | Açaí berry |
|  | Arroz e feijão | Black bean stew with rice |
|  | Pastel | Crispy turnover |
|  | Tapioca | Tapioca pancake |
|  | Caipirinha | Cocktail with cachaça |
|  | Pão de queijo | Cheese bread balls |
|  | Coxinha | Crunchy drop-shaped dough filled with chicken |
|  | Brigadeiro | Sweet chocolate balls |
|  | Feijoada | Stew with black beans and meat |
| Mexico |  |  |
|  | Chile relleno | Stuffed sweet chili |
|  | Atole | Warm maize beverage |
|  | Aguas frescas | Light sweet juice with ice |
|  | Barbacoa | Steamed meat |
|  | Elotes preparados | Corncob with mayonnaise and chili |
|  | Pulque | Fermented maguey juice |
|  | Quesadillas | Filled half-moon corn tortilla |
|  | Antojitos dorados | Deep-fried coarse milled corn tortilla with meat and sauce |
|  | Tequila | Tequila |
|  | Enchiladas | Filled soft corn tortilla |
|  | Antojitos no dorados | Coarse milled corn tortilla with meat and sauce |
|  | Pozole | Soup with maize and meat |
|  | Tamales | Filled steamed maize dough |
|  | Tacos | Soft corn tortilla with meat and veggies |
|  | Mole | Main dishes with spicy sauce |
| Chile |  |  |
|  | Porotos con riendas | Soup with beans and spaghetti |
|  | Chicha | Fermented grape juice |
|  | Porotos con maíz | Stew with beans and maize |
|  | Chilean wine | Chilean wine |
|  | Sopaipillas | Deep-fried dough |
|  | Drinks with pisco | Drinks with “pisco” (grape brandy beverage) |
|  | Charquicán | Stew with meat (jerky) and veggies |
|  | Mote con huesillos | Husked wheat with peaches |
|  | Humitas | Filled corn husks |
|  | Empanadas | Chilean turnover |
|  | Cazuela | Soup with meat and veggies |
|  | Pastel de choclo | Beef and corn puree casserole |
| China |  |  |
|  | 鱼香肉丝 (Yu Xiang Rou Si) | Yuxiang shredded pork |
|  | 白酒, 烧酒 (Baijiu, Shaojiu) | Distilled alcoholic beverage |
|  | 油条 (Youtiao) | Chinese crullers |
|  | 月饼 (Yue bing. mooncake) | Mooncake |
|  | 宫保鸡丁 (Kung pao chicken) | Spicy chicken with peanuts |
|  | 粽子 (Zongzi) | Chinese sticky rice pyramid |
|  | 汤圆（元宵） (Tang yuan) | Glutinous rice balls |
|  | 凉皮 (Liang pi) | Cold skin noodles |
|  | 肉夹馍 (Rou jia mo) | Chinese hamburger |
|  | 烤鸭 (Kao ya) | Peking duck |
|  | 火锅类食品 (Chinese hotpot) | Chinese hotpot |
|  | 包子 (Baozi) | Baozi |
|  | 饺子 (Jiaozi) | Dumpling |
| Japan |  |  |
|  | すき焼き(Sukiyaki) | Japanese hot pot |
|  | 米菓(Beika) | Rice based snacks |
|  | 肉じゃが(Nikujyaga) | Stew with potatoes, meat and onion |
|  | おにぎり(Onigiri) | Rice balls with meat/vegetables/fish/seaweed |
|  | 刺身(Sashimi) | Sashimi |
|  | 納豆(Natto) | Fermented soybean |
|  | 漬物(Tsukemono) | Pickled vegetables |
|  | うどん(Udon) | Udon noodles (made from wheat flour) with dipping sauce/broth |
|  | 蕎麦(そば,soba) | Soba noodles (made from buckwheat flour usually mixed with wheat flour) with dipping sauce/broth |
|  | 天ぷら(Tempura) | Deep-fried seafood/meat/vegetables with tempura batter |
|  | 日本茶(Nihoncha) | Japanese tea |
|  | 日本酒(Nihonshu) | Sake (fermented rice beverage) |
|  | 味噌汁(Misoshiru) | Miso soup |
|  | 寿司(Sushi) | Sushi |
| Indonesia |  |  |
|  | Kerupuk | Deep-fried crackers |
|  | Wedang | Ginger syrup-based preparations |
|  | Soto | Indonesian soups |
|  | Keripik | Indonesian chips |
|  | Nasi dishes | Dishes containing rice, meat and vegetables |
|  | Rendang | Slow-cooked spicy beef |
|  | Cendol/dawet | (Iced) beverages with cendol (small pieces of rice noodle) |
|  | Aneka gorengan | Deep fried fritters |
|  | Kue basah (savoury) | Savoury moist snacks |
|  | Kue basah (sweet) | Sweet moist snack |
